# Case Report: Primary Cutaneous Aspergillosis in a Neutropenic Child with Pulmonary Dissemination: A case report

**DOI:** 10.12688/f1000research.178649.1

**Published:** 2026-03-30

**Authors:** Yasmine KALBOUSSI, Wafa CHENBAH, Hamed CHOUAIEB, Nour GAALOUL, Samar ISMAIL, Monia GUERMAZI, Mariem BEN TICHA, Imene KHAMMARI, Yosra BEN YOUSSEF, Akila FATHALLAH

**Affiliations:** 1 Laboratory of Parasitology - Mycology, Farhat Hached University Hospital of Sousse, Sousse, Sousse, Tunisia; 2 University of Monastir Faculty of Medicine of Monastir, Monastir, Monastir, Tunisia; 3 Department of Hematology, Farhat Hached University Hospital of Sousse, Sousse, Sousse, Tunisia; 4 University of Sousse Faculty of Medicine of Sousse, Sousse, Sousse, Tunisia; 5 Department of Infectious diseases, Farhat Hached University Hospital of Sousse, Sousse, Sousse, Tunisia

**Keywords:** Aspergillosis, cutaneous aspergillosis, neutropenia, invasive pulmonary aspergillosis, case report

## Abstract

**Introduction:**

Invasive aspergillosis is a severe infection that usually affects immunocompromised patients. Primary cutaneous involvement is a rare presentation and presents a challenge for early diagnosis. We report a case of primary cutaneous aspergillosis (PCA) in an immunocompromised patient with no evident prior skin trauma and with pulmonary dissemination.

**Case report:**

We report the case of primary cutaneous aspergillosis involving the left ankle in a 5-year-old girl with no history of preceding trauma. The patient was undergoing chemotherapy for lymphoblastic leukemia. *Aspergillus* hyphae were identified on skin biopsy. Cultures grew *Aspergillus flavus.* The diagnosis of cutaneous aspergillosis enabled the diagnosis of probable pulmonary aspergillosis, although there was no mycopathological proof of lung infection. The patient was treated with initial Amphotericin B followed by Voriconazole with complete skin and respiratory response.

**Conclusion:**

This case underscores the critical need to consider cutaneous aspergillosis in immunocompromised patients with necrotic skin lesions— even in the absence of obvious trauma—as prompt diagnosis and treatment are vital to prevent dissemination and and improve outcomes.

## Introduction

Invasive aspergillosis is a severe and potentially fatal infection usually affecting immunocompromised patients. ^1, 2^ Pulmonary involvement is the predominant presentation, whereas cutaneous localization is much less frequent. This can develop as a primary infection, usually arising from direct inoculation of the skin ^3^ or occur as part of a disseminated infection from the lung. ^1^ The true incidence of primary cutaneous aspergillosis (PCA) among immunocompromised patients is not well established but seems to be rising, possibly as a result of better recognition and the increasing number of immunocompromised individuals. ^4, 5^ The major limitation in the management of these infections is the challenge of early diagnosis. We report a rare case of primary cutaneous aspergillosis (PCA) caused by *Aspergillus flavus* in a neutropenic and immunocompromised 5 year- old patient with no evident prior skin trauma.

## Case report

**Figure 1.**
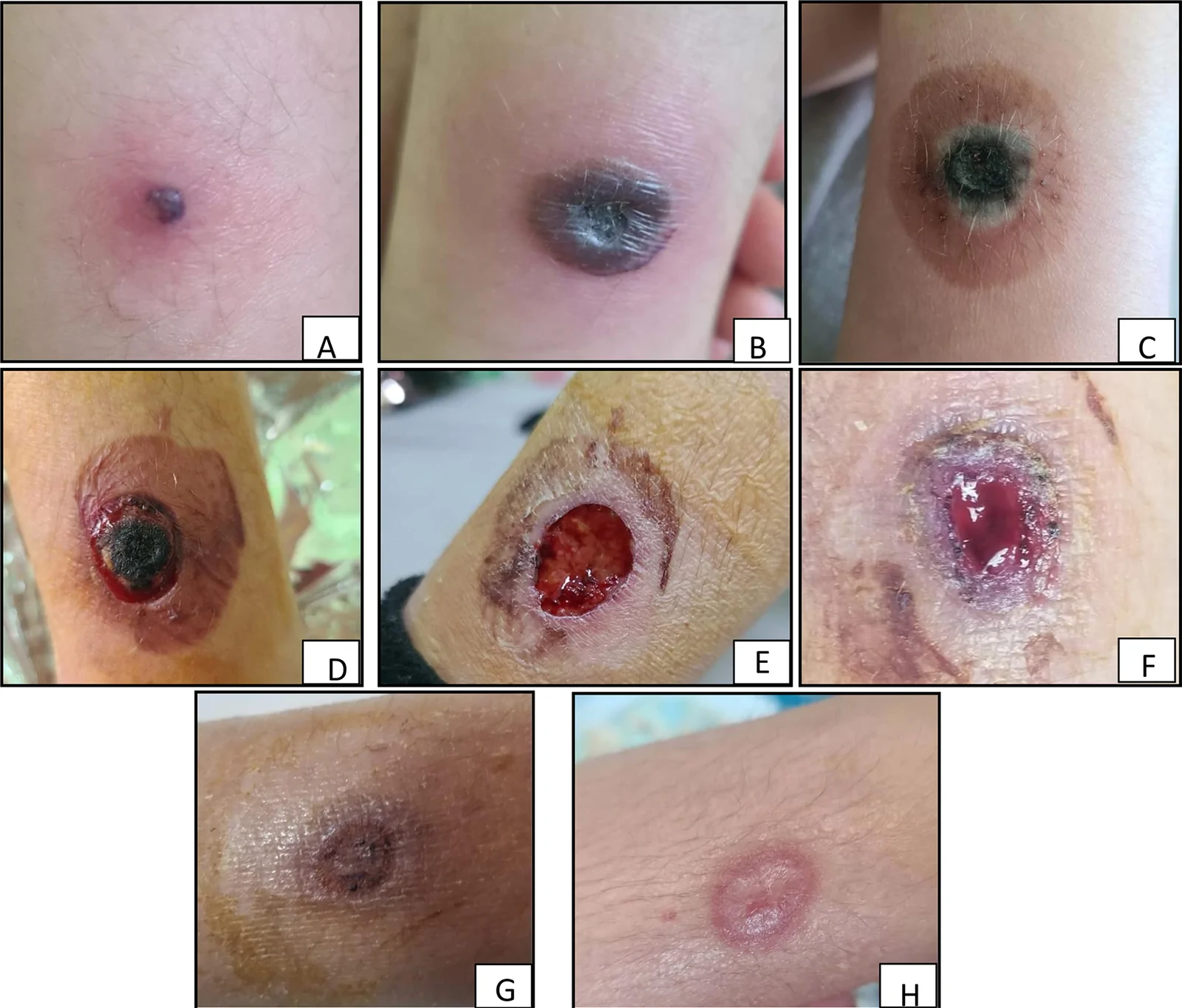
Primary cutaneous aspergillosis of the left ankle: A, Initial skin lesion; B, Day 4; C, Day 5; D, Two days after Amphotericin; E, Day 7 of Amphotericin B; F, Lesion aspect after Voriconazole switch; G and H, Complete skin remission under Voriconazole.

A 5-year-old girl was admitted to the Haematology department of Farhat-Hached hospital (Sousse, Tunisia) in September 2024 for acute lymphoblastic leukemia. She received chemotherapy consisting of anthracyclines, L-asparaginase, cyclophosphamide, methotrexate and long-term corticosteroid therapy. In April 2025, she was admitted to receive consolidation chemotherapy. Seven days after the last dose, she developed high-grade fever (40 °C), concomitantly with the appearance of a small 5-mm black spot on her left ankle ( Figure 1). The patient was neutropenic, with an absolute neutrophil count <500/mm ^3^ for 5 days. The initial infectious workup was negative. She was started on broad-spectrum antibiotics made of Piperacillin-Tazobactam and Vancomycin. Three days later, the patient still febrile and the skin lesion grew larger about 1.5 cm, becoming swollen with a crusted center and red surrounding skin. Antibiotics were switched to Imipenem and Ciprofloxacin with no improvement.

When a mild cough appeared few days after, a full-body CT scan was done. It showed nodules and lung infiltrates with a halo sign highly suggestive of angio-invasive pulmonary aspergillosis. Intravenous amphotericin was started immediately. Within 12 hours, she became afebrile for the first time. The skin lesion on her ankle evolved: it became itchy, developed a necrotic center with a purplish halo, and later the center dried out, with a hemorrhagic edge and ulceration. Eventually, the necrotic center detached from the lesion. This necrotic tissue was sent to the mycology laboratory for analysis.

**Figure 2.**
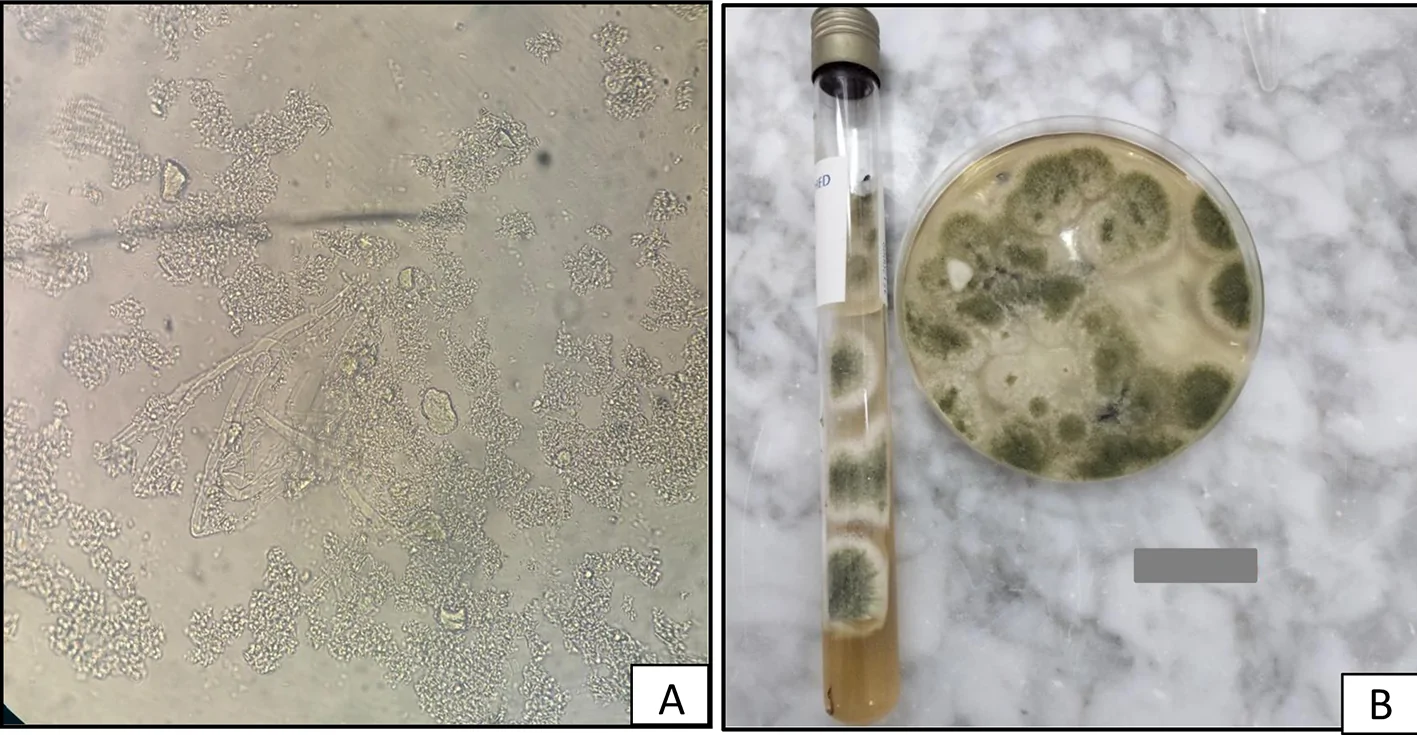
Mycological findings: A, large, septate, and irregular hyphae with acute-angle branching on Direct Examination suggestive of *Aspergillus* (X100); B, *Aspergillus flavus* growing on culture.

Microscopic examination revealed large, septate, and irregular hyphae with acute-angle branching, suggestive of *Aspergillus* ( Figures 2). The specimen was cultured on Sabouraud dextrose agar supplemented with chloramphenicol (SC) and incubated at 30 °C. Fungal growth, 4 days after incubation, was consistent with *Aspergillus.* Identification using the Vitek MS PRIME (bioMérieux, France), yielded *Aspergillus flavus.* Antifungal susceptibility testing was carried out using the MIC Test Strip method (Liofilchem, Roseto degli Abruzzi, Italy). The minimum inhibitory concentrations (MICs) were as follows: 0.75 mg/L for Amphotericin B and 0,38 mg/L for Voriconazole. No respiratory specimens were submitted for mycological examination. Concurrently, the serum galactomannan index was measured using the Platelia *Aspergillus* enzyme immunoassay (Bio-Rad, France), yielding a positive result with an index value of 0.57.

Diagnosis of PCA with probable pulmonary dissemination was confirmed. Based on these findings, antifungal therapy was switched to Voriconazole, leading to complete resolution of the cutaneous lesions. The patient was subsequently discharged with a favorable outcome. With regular follow-up and local wound care, she achieved full recovery, and no relapse was observed at 6-month follow-up.

## Discussion

We report a rare case of primary cutaneous aspergillosis (PCA) caused by *Aspergillus flavus* in a neutropenic patient with no evident prior skin trauma. The infection occurred following consolidation chemotherapy for acute lymphoblastic leukemia, during the aplastic phase, and subsequently progressed to invasive pulmonary aspergillosis. This case demonstrates the rapid progression of an initially inconspicuous cutaneous lesion to a severe necrotizing infection. It underscores the importance of careful clinical evaluation, as seemingly minor skin findings may represent an entry point for invasive fungal disease. Clinicians should maintain a high index of suspicion for primary cutaneous aspergillosis in neutropenic patients. Therefore, even a localized skin lesion requires aggressive systemic antifungal therapy to prevent dissemination.

Pediatric patients undergoing chemotherapy for hematological malignancies as illustrated by this 5-year-old girl, represent a high-risk cohort for invasive fungal infections. ^1, 2^ The most associated disorders in children are leukemias and lymphomas. ^3^ However, some cases have been reported with immunocompetent patients. ^4^ Intensive regimens, including anthracyclines and cyclophosphamide, induce profound and prolonged neutropenia, which is the major risk factor for invasive aspergillosis. ^5^ Corticosteroids, a key component of leukemia protocols, further impair immune defenses by suppressing macrophage and neutrophil function, crippling the host’s ability to contain fungal invasion. Consequently, this immunocompromised state, defined by cytotoxic and steroid-induced deficits, creates a perfect environment for invasive fungal diseases.

Cutaneous aspergillosis can occur either as a primary infection or as a secondary manifestation. ^6, 7^ Primary cutaneous aspergillosis (PCA) typically results from the direct inoculation of spores into the skin via breaches in barrier integrity, such as at catheter insertion sites, trauma wounds, or beneath occlusive dressings. In contrast, secondary cutaneous involvement occurs almost exclusively through hematogenous dissemination ^7, 8^ from a deep-seated focus, most commonly the lung. This form is associated with the angioinvasive behavior of *Aspergillus* species. Determining whether the infection is primary or secondary to a primary site, such as the lungs, is crucial for guiding treatment. ^9^ In our case, the initial isolated cutaneous lesion, which appeared concomitantly with the onset of fever **,** and the absence of radiological lung abnormalities at presentation, strongly supports the diagnosis of primary cutaneous aspergillosis with subsequent pulmonary dissemination. This occurred in the absence of any clinically apparent skin injury, which, to our knowledge, appears to be exceptional. However, a minor or unnoticed breach in the skin barrier cannot be excluded. This case highlights that primary cutaneous aspergillosis should be considered even in the absence of evident skin trauma.

PCA can have different presentations: erythematous macules and papules with pain and itching, necrotizing skin lesions, hemorragic bullas, ulcerations with central necrosis or violaceous nodules. ^10^ The variety of presentations and the non-typical form of lesions lead to an underdiagnosis of PCA and emphasize on the importance of mycological examination in order to start antifungal treatment and avoid the dissemination.

The definitive diagnosis of cutaneous aspergillosis relies on examination and culture of a deep tissue biopsy, as superficial samples are often inadequate. ^11^ Direct examination typically reveals septate hyphae with acute-angle branching, suggestive of *Aspergillus*, though not pathognomonic, as similar hyaline molds like *Fusarium* must be excluded. ^8^ While *A. fumigatus* predominates in invasive aspergillosis overall, accounting for approximately 53% of pediatric cases in the largest multicenter study, ^1^ *A. flavus* is notably prevalent in primary cutaneous infections (PCA), accounting for a significant proportion of cases. ^6, 12^ Our case aligns with this epidemiological profile for PCA. Serological biomarkers, such as serum galactomannan (GM), provide valuable adjunctive evidence. In pediatric patients, GM assay offers good sensitivity and specificity, and a positive result in a high-risk clinical context strongly supports the diagnosis of invasive disease. ^8^ In this case, the positive GM antigenemia, concomitant with the cutaneous biopsy results and pulmonary imaging, was instrumental in confirming disseminated infection.

According to the EORTC/MSGERC criteria, ^13^ our patient fulfilled the definition of proven invasive cutaneous aspergillosis, based on the demonstration of septate hyphae in direct examination from a deep skin biopsy and confirmatory culture yielding *Aspergillus.* However, imaging alone is insufficiently specific for diagnosing pulmonary aspergillosis, as current guidelines require microbiological evidence for a probable infection.

The antifungal susceptibility testingc onfirmed a fully susceptible profile, with a notably low Voriconazole MIC of 0.38 mg/L, strongly justifying its use as primary therapy according to EUCAST guidelines. The patient’s sequential antifungal regimen—initial Amphotericin B followed by Voriconazole—is a common clinical strategy for managing suspected invasive fungal infections. ^14, 15^ Most patients treated with one or the other had full recovery. ^16^ However, this approach requires careful consideration due to on going debates about potential antagonistic interactions between the two drug classes. ^17^ Surgical debridementand oral Itraconazole are also therapeutic options with extended lesions. ^18^

## Conclusion

This clinical case highlights the importance of considering cutaneous aspergillosis in immunocompromised patients presenting with skin lesions that progress to necrosis, even in the absence of trauma history. Particular attention must be paid to such lesions, as prompt diagnosis and treatment are crucial to prevent dissemination and reduce infection-related mortality.

## Consent to publish

A written consent was provided and signed by the patient’s parent, including the authorization for publishing clinical details and/or clinical images.

## Data Availability

The CARE chechlist is publicly available at Zenodo. ^19^ Title: Completed CARE checklist associated with the manuscript entitled “Primary Cutaneous Aspergillosis in a Neutropenic Child with Pulmonary Dissemination: A case report”. DOI: https://doi.org/10.5281/zenodo.18860965. ^19^ License: CC0 1.0.
