# Supplementary material for: Case Report: Primary Cutaneous Aspergillosis in a Neutropenic Child with Pulmonary Dissemination: A case report
Source: F1000Res. 2026 Jun 2;15:454. Originally published 2026 Mar 30. [Version 2] doi: 10.12688/f1000research.178649.2 (PMC13555018; doi:10.12688/f1000research.178649.2)
Supplement: Peer review history

# Reviewer report: Reviewer response for version 2

**DOI:** 10.5256/f1000research.202207.r500373

**Published:** 2026-07-24

**Authors:** Christopher Heath

**Affiliations:** 1 Department of Medicine, University of Western Australia, Perth, Western Australia, Australia; 2 Infectious Diseases & Clinical Microbiology, Fiona Stanley Hospital, Murdoch, Western Australia, Australia

**Comments to the Authors:**

 This is an interesting case report that has a number of important take-away messages. However, there are a number of constraints with current version of the manuscript that should be addressed by the authors.

**Major comments: **

1. **Title of manuscript: Case report: page 3 of manuscript: **

In my opinion, there are multiple issues with the current manuscript. In essence, it lacks enough precision on a number of key points.

 Firstly, although the diagnosis of primary cutaneous aspergillosis (PCA) seems quite likely; was histopathology performed on a deep skin biopsy taken in this case? If so, did it show true tissue invasion, with the presence of hyphae that are accompanied by evidence of infection associated tissue damage, as per EORTC/MSG diagnostic definitions (Donnelly JP, et al. CID 2020)? If so, please provide this information for the case report. If no deep skin biopsy was performed for histopathology, then it is incorrect to use the term ‘proven’ cutaneous aspergillosis. In this case, the best synopsis is to label this case as: probable cutaneous aspergillosis.

 Secondly, mycology seems to be derived from the sloughed centre of a necrotic skin lesion. In essence, while cutaneous aspergillosis seems quite likely in this case, the use of ‘sloughed necrotic tissue’ for mycological testing, does not meet the mycological EORTC/MSG diagnostic criteria (Donnelly JP, et al. CID 2020). Basically, a deep skin biopsy specimen is required to be obtained from a sterile procedure from a normally sterile site.

 Thirdly, the use of the term PCA and the use of dissemination in the title. Virtually all cases of human invasive aspergillosis are acquired first from the respiratory tract. Whilst PCA does occur, it is very rare, and in my clinical experience is almost invariably associated with local trauma or another local explanation. No reason for the patients’ skin lesion is provided, making PCA relatively unlikely, in my clinical experience.

 Fourthly, whilst there is some evidence for pulmonary aspergillosis it is likely to be ‘possible IA’ based on EROCT/MSG criteria (Donnelly JP, et al. CID 2020). Further, the detection of lung pathology by CT chest, after the presentation of the skin lesion, in no way proves primary cutaneous aspergillosis (PCA). Indeed, radiological lung signs are often clinically silent and require pro-active screening by HRCT, in high-risk patients particularly in the setting of unresolved febrile neutropenia. The currently reported CT findings are open to interpretation; the report states that the CT showed: ‘lung infiltrates with halo sign’. However, the halo sign can only be specifically associated with one or more of the lung nodules. Further description of the lung nodules needs to be provided; viz., size, location and whether there was the halo sign associated with one or more of the nodules. Was the patient still neutropenic 7 days after the onset of the cutaneous lesion (i.e., at the time of this CT chest)? Further description of the lung infiltrates should also be provided for the reader. Alternatively, high-quality image(s) of the HRCT scan showing the key lung pathology should be provided, to enable this to be adjudicated.

 In summary, you need to precisely follow the EROCT/MSG diagnostic criteria for IA (2020). I would therefore recommend referring to this case as: A case of probable cutaneous *A. flavus* complex (please, see below) infection, with possible pulmonary aspergillosis.

  

1. **Identification of the isolate as *A. flavus*:  **The current wording implies that *A. flavus* *sensu stricto* was identified. However, the organism was identified using Vitek MS PRIME which uses MALDi-TOF proteomic technology. That does not have the capability to diagnose to an unequivocal specific species level. For example, the paper by Dutkiewicz M, et al. JCM **2025**, showed that species identification rates for Vitek MS PRIME were only 77%. The manifestations of the colony and slide culture appear to show features consistent with *A. flavus*, however, the microscopy is of a very poor quality. I can’t clearly see all of the features described. Can it be improved? Was a slide mount with lactophenol blue microscopy performed?  Further, full speciation to the level of *A. flavus sensu stricto* requires nucleotide sequencing of the ITS regions of the ribosomal 18S DNA gene, and partial sequencing of either the β-tubulin and/or calmodulin genes. This matter is important, as there is at least one published case of *A. tamarii* (also part of the *A. flavus* complex) causing cutaneous aspergillosis. In summary, I would recommend referring to this isolate as: *A. flavus* complex.

**Minor comments:**

1. **Introduction: page 3:** Rising incidence of PCA. ‘Seems to be rising’ is overly vague and not linked to any specific data to support this statement. Is there specific data showing a rising incidence rate? Revise and/or remove.

 

1. **Case report: page 3, paragraph 1: **The patient received anthracyclines. Does that mean the patient received more than one anthracycline? If not, please specify the anthracycline prescribed.

 

1. **Case report: page 3, paragraph 1: **The term: ‘blastic cells’ should be simplified to ‘blasts’.

 

1. **Case report: page 3, paragraph 1: **The duration of dexamethasone. ‘During 5 days…’, is a little unclear as to the duration &/or timing of steroids in the chemotherapy regimen. It would be better to say that: ‘dexamethasone was given over 5 days.’

 

1. **Case report: page 3: **Lesion size is initially reported as: ‘small 5-mm’. The size alone stated as ‘5-mm’ will suffice. Lesion appearance is described as a: ‘black spot’. Please use appropriate dermatological terms. Lesion size is later reported as: ‘larger about 5.1.-cm’. Can there be greater precision on the lesion size, please. Finally, the skin is described as: ‘red surrounding skin’. Suggest, ’Skin erythema’, as dermatologically more correct terminology.

 

1. **Case report: page 3, second paragraph: **The word ‘days’, is presumably missing after the word seven. Please correct. The CT imaging of the lungs: Was the lung imaging a high-resolution CT (HRCT) chest? This point should be clarified in the manuscript.

 

1. **Case report: page 3, third paragraph: **The word ‘ *Aspergillus’* in this context should be followed by the term ‘species’ or ‘spp.’. Also recommend use of the taxonomically correct term: *A. flavus* complex. The Liofilchem MIC Test Strips (MTS™) for fungi are only CE IVD and RUO, in the EU and the US, respectively. That is, they E-test strips are not included for fungi in the EUCAST methods recommended – that is, only microdilution plates are described. The used of graduated test strips for *Aspergillus* spp. have been reported as having only ‘reasonable performance’ (Idelevich EA, et al. Diag Microbiol Infect Dis, 2018, 91(1):52-54.), and the results reported here will need to be interpreted more cautiously in the revised Discussion.

 

1. **Case report: page 5, 1 ^st^. paragraph: **The EORTC/MSG guidelines (Donnelly JP, CID 2020) for reporting a positive GM EIA result from a single serum or plasma sample is an optical density (OD) (also referred to as, optical index) of  ≥1.0. Whilst the manufacturers recommend a cut-off value of ≥0.5; this is not a true positive result in terms of agreed international guidelines (Donnelly JP, et al). The absence of any fungal respiratory cultures the level for mycological evidence the diagnosis of invasive pulmonary aspergillosis can only be rated as ‘possible’.

 

1. **Discussion: page 5, 1 ^st^. paragraph: **Please change the first sentence given the above comments. We also have no definite evidence that her probable cutaneous aspergillosis led to possible IPA. Please revise to more appropriate conclusions.

 

1. **Discussion: page 5, 2 ^nd^. paragraph: **Regarding the US-based Kids’ Inpatient Database and the reference appears to be missing (new reference). Also, regarding effects of corticosteroids: they do not lead to a: ‘crippling’ of the immune system. Please use more appropriate immunological term.

 

1. **Discussion: page 5, 3 ^rd^. paragraph: **In discussing the forms of cutaneous aspergillosis, please be less dogmatic that this patient had proven primary cutaneous aspergillosis (PCA). I also disagree on the further point that states that PCA and secondary pulmonary aspergillosis are treated different to other forms of invasive aspergillosis. No guidelines make these distinct treatment recommendations. Therefore, please be less dogmatic about this being ‘crucial for guiding treatment’. No radiological lung imaging is reported at baseline. Even if performed at baseline, HRCT changes in the lungs can take many days to evolve pathology. The point made about this case being ‘exceptional’ in the absence of skin injury should be removed. The last 2 sentences of the paragraph seem to repeat themselves. Please reword, and/or remove one sentence.

 

1. **Discussion: page 5, 4 ^th^. paragraph: **The Discussion should reflect on the range of clinical phenotypes associated with cutaneous aspergillosis, rather than specifically PCA.

 

1. **Discussion: page 5, 5 ^th^. paragraph: **The Discussion herein highlights again why a definitive diagnosis of cutaneous aspergillosis cannot be made in this case, as a deep tissue biopsy was not performed. Please revise the wording used, to be less dogmatic on this point. The serological biomarker GM EIA was negative by internationally agreed guidelines (Donnelly JP, et al. CID 2020) and therefore should not be offered as ‘valuable adjunctive evidence’. Moreover, the performance of the GM EIA sensitivity and specificity in paediatric patients, are in general much less reliable than the results obtained in high-risk adult patients with haematological malignancies.

 

1. **Discussion: page 6, 1 ^st^. paragraph: **The result of the GM EIA assay, by itself, does not confirm or refute the diagnosis of disseminated aspergillosis. Please remove.

 

1. **Discussion: page 6, 2 ^nd^. paragraph: **The Discussion herein again highlights why a definitive diagnosis of cutaneous aspergillosis cannot be made in this specific case. 

 

1. **Discussion: page 6, 3 ^rd^. paragraph: **The EUCAST guideline AFST results do not certify the specific use of one antifungal over another. A voriconazole MIC of 0.38 mg/L, is likely within the wild-type range for this organism, but that does not mandate the use of voriconazole. Likewise, the same likely applies to the Amphotericin B (AmB) MIC results of 0.75 mg/L. Guidelines recommend voriconazole as primary therapy. Indeed, in contrast to the manuscript that refers to: “amphotericin B followed by voriconazole”, this is not specifically mentioned as a standard treatment strategy in current guidelines. Voriconazole has demonstrated efficacy as first-line therapy for invasive aspergillosis (IA) in RCTs and is strongly supported by high-quality evidence for both IV and/or PO therapy. In the IDSA Guidelines from 2016 (Paterson TF, et al. 2016, CID) quoted by the authors the wording is as follows for IA: “recommend voriconazole as primary therapy for IA (strong recommendation; moderate-quality evidence). Lipid formulations of AmB are *reserved for those intolerant or refractory to voriconazole* [my italics added] (strong recommendation; moderate-quality evidence). Contrary to this manuscript, sequential therapy from different antifungal classes is not given a specific recommendation in the IDSA, 2016, Guidelines. Moreover, in the Australian Guidelines there is again a preference for voriconazole as the primary therapy (strong recommendation, Level I evidence (Douglas AP, et al IMJ, 2021). Conversely, these guidelines state the use of Amphotericin B in this clinical context, is only rated as moderate recommendation, Level II evidence (Douglas AP, et al IMJ, 2021).

Essentially, regarding this paragraph: I suggest a rewording of this section of the manuscript in keeping with both the evidence from published studies/guidelines together with the pragmatics of individual patient care.

 Lastly, the comment about possible antagonistic interactions between two drug classes, quotes a study by Vazquez JA, et al. 1996, that reports only on azole vs. amphotericin B drug-drug interactions for treatment of *Candida albicans*. This is not an appropriate reference, for a case of aspergillosis.

  

1. **References: pages 6-7: **Many of the references are old and/or do not directly report on the question/comments made within this manuscript. Suggest where possible use more modern papers of higher quality &/or use more specific wording in the final manuscript to link this phrasing to each cited reference.

Are enough details provided of any physical examination and diagnostic tests, treatment given and outcomes?

Partly

Is the case presented with sufficient detail to be useful for other practitioners?

Yes

Is sufficient discussion included of the importance of the findings and their relevance to future understanding of disease processes, diagnosis or treatment?

Partly

Is the background of the case’s history and progression described in sufficient detail?

Partly

Reviewer Expertise:

Clinical and Laboratory Mycology, Infections in the Immunocompromised Host (e.g., BM transplant medicine), Infections after solid organ transplantation, major burns and major trauma, and Infections in the ICU.

I confirm that I have read this submission and believe that I have an appropriate level of expertise to confirm that it is of an acceptable scientific standard, however I have significant reservations, as outlined above.

## References

[1] : Comparison of MALDI-TOF MS instruments and databases for the identification of uncommon yeasts,Aspergillus spp. and rare filamentous fungi. *Journal of Clinical Microbiology* .2025;63(6) : 10.1128/jcm.01612-24 10.1128/jcm.01612-24 PMC1215331540372046

[2] : Consensus guidelines for the diagnosis and management of invasive aspergillosis, 2021. *Internal Medicine Journal* .2021;51(S7) : 10.1111/imj.15591 143-176 10.1111/imj.15591 34937136

# Reviewer report: Reviewer response for version 2

**DOI:** 10.5256/f1000research.202207.r490227

**Published:** 2026-07-21

**Authors:** Rim Ben Abdallah

**Affiliations:** 1 Institut Pasteur de Tunis, Place Pasteur, Tunisia

Thank you for your careful revision of the manuscript. Most of the comments have been adequately addressed, and the manuscript has been substantially improved. However, a few minor editorial comments remain unaddressed:

 1. Abstract       

 - and and improve outcomes” → remove duplicate “and”.

 Please remove the duplicated word "and" in the phrase "and and improve outcomes" in the Abstract.

 2. Introduction

 'No evident prior skin trauma' is an important point, but it could be better contextualised by briefly mentioning that PCA is usually associated with skin breaches caused by catheters, dressings or trauma, which makes this case unusual.

 The sentence "PCA is typically associated with breaches in skin integrity caused by catheters, dressings, or trauma, making the present case particularly unusual" may be better placed immediately before the sentence introducing the case report. This would provide a more logical progression from the general characteristics of primary cutaneous aspergillosis to the specific originality of the reported case.

 - 5 year- old patient” → correct spacing: 5-year-old patient

 This comment has not been addressed. Please correct "5 year- old patient" to "5-year-old patient" throughout the manuscript.

 - The abbreviation 'PCA' should be used throughout the remainder of the manuscript after the first definition of primary cutaneous aspergillosis.

 This comment has not been addressed. After the first definition of primary cutaneous aspergillosis, please use the abbreviation "PCA" consistently throughout the remainder of the manuscript.

Are enough details provided of any physical examination and diagnostic tests, treatment given and outcomes?

Partly

Is the case presented with sufficient detail to be useful for other practitioners?

Partly

Is sufficient discussion included of the importance of the findings and their relevance to future understanding of disease processes, diagnosis or treatment?

Partly

Is the background of the case’s history and progression described in sufficient detail?

Yes

Reviewer Expertise:

parasitology-mycology

I confirm that I have read this submission and believe that I have an appropriate level of expertise to confirm that it is of an acceptable scientific standard.

# Reviewer report: Reviewer response for version 2

**DOI:** 10.5256/f1000research.202207.r490228

**Published:** 2026-06-09

**Authors:** Rim Rakkez

**Affiliations:** 1 Hematology, University Hospital of Martinique (Ringgold ID: 55525), Fort-de-France Bay, Fort-de-France, Martinique

The revised version of the article was perfect.

Are enough details provided of any physical examination and diagnostic tests, treatment given and outcomes?

Yes

Is the case presented with sufficient detail to be useful for other practitioners?

Yes

Is sufficient discussion included of the importance of the findings and their relevance to future understanding of disease processes, diagnosis or treatment?

Yes

Is the background of the case’s history and progression described in sufficient detail?

Partly

Reviewer Expertise:

acute leukemias, myeloid malignancies

I confirm that I have read this submission and believe that I have an appropriate level of expertise to confirm that it is of an acceptable scientific standard.

# Reviewer report: Reviewer response for version 1

**DOI:** 10.5256/f1000research.197060.r481919

**Published:** 2026-05-18

**Authors:** Rim Rakkez

**Affiliations:** 1 Hematology, University Hospital of Martinique (Ringgold ID: 55525), Fort-de-France Bay, Fort-de-France, Martinique

I would like to thank the authors for this article, which is very interesting scientifically and written in clear, fluid English. However, I have a few comments that require minor revisions.

 First, in the case report, you mentioned that the patient was admitted for consolidation chemotherapy. Could you specify the protocol used for ALL treatment? What was his hematological status?

 Second, Could you indicate after how many days of neutropenia did the cough appear? were the antifungal biomarkers performed ? you did mention the cutaneous lesion and its link to fever, but you did not specify the initial imaging done at the start, which showed no pulmonary lesions. This should be clearly stated in the case report.

 Finally, in the discussion, I think it is better to go straight to the core of the topic and avoid a review of the case. In addition, the epidemiology section on this cutaneous aspergillus infection should be slightly expanded to include whether many cases have been described in leukemic children. In conclusion, this case report demonstartes that one should not hesitate to biopsy any skin lesion mainly in the absence of infectious or microbiological documentation in a febrile neutropenic patient

Are enough details provided of any physical examination and diagnostic tests, treatment given and outcomes?

Yes

Is the case presented with sufficient detail to be useful for other practitioners?

Yes

Is sufficient discussion included of the importance of the findings and their relevance to future understanding of disease processes, diagnosis or treatment?

Yes

Is the background of the case’s history and progression described in sufficient detail?

Partly

Reviewer Expertise:

acute leukemias, myeloid malignancies

I confirm that I have read this submission and believe that I have an appropriate level of expertise to confirm that it is of an acceptable scientific standard.

# Response

**Published:** 2026-05-20

**Authors:** Yasmine Kalboussi

**Affiliations:** Parasitology-Mycology, Farhat Hached University Hospital of Sousse, Sousse, Sousse, Tunisia

Dear Reviewer,

 We sincerely thank you for your careful re-evaluation of our manuscript and for your continued valuable comments. We greatly appreciate the time and effort you have dedicated to improving the quality of our work.

 We have carefully addressed all of your remarks and revised the manuscript accordingly. These modifications have further contributed to enhancing the clarity and accuracy of the article.

 Please find below our point-by-point responses to your comments:

- **Comment :** First, in the case report, you mentioned that the patient was admitted for consolidation chemotherapy. Could you specify the protocol used for ALL treatment? What was his hematological status?

**Response:** Thank you for this relevant comment. We added ‘The patient was treated according to EORTC protocole (Very High-Risk aka VHR group) and received Chemotherapy VANDA consisting of anthracyclines, L-asparginase , cyclophosphamide, methotrexate and corticosteroids (12mg of dexamethasone per day during 5 days). She had a cytological remission: 3% Blastic cells but a positive minimal residual disease (MRD) on the Last Bone Marrow assessement.’

  

- **Comment:** Second, could you indicate after how many days of neutropenia did the cough appear? were the antifungal biomarkers performed ? you did mention the cutaneous lesion and its link to fever, but you did not specify the initial imaging done at the start, which showed no pulmonary lesions. This should be clearly stated in the case report.
**Response:** Thank you for this insightful comment. We confirm that the cough appeared seven days after the onset of the cutaneous lesion during the period of neutropenia, and this timing has been clarified in the revised manuscript. Regarding the initial evaluation, no baseline chest imaging was performed at presentation prior to the development of respiratory symptoms. In addition, no respiratory sampling was carried out. Serum galactomannan antigen was measured and showed a borderline positive result, as previously reported, and was interpreted with caution in the overall diagnostic assessment

 

- **Comment :** Finally, in the discussion, I think it is better to go straight to the core of the topic and avoid a review of the case. In addition, the epidemiology section on this cutaneous aspergillus infection should be slightly expanded to include whether many cases have been described in leukemic children.

**Response:** Thank you for this helpful suggestion. We have revised the Discussion section to remove redundant case descriptions and focus more directly on the core scientific message of the report, as recommended.

 In addition, we have expanded the epidemiological section. We added ‘The population-based incidence of invasive aspergillosis (IA) in children remains unknown and is likely to vary between healthcare settings and countries. However, data from the Kids’ Inpatient Database, a large representative dataset of hospitalizations in the United States, reported an annual incidence of 437 cases per 100,000 (0.4%) hospitalized immunocompromised children in 2000.’

 We hope that the revised version adequately addresses your concerns and meets the journal’s expectations.

 Thank you again for your constructive feedback.

 Sincerely,

 Yasmine Kalboussi

# Response

**Published:** 2026-06-02

**Authors:** Rim Rakez

Thank you for the revision you made to the article!

# Reviewer report: Reviewer response for version 1

**DOI:** 10.5256/f1000research.197060.r472149

**Published:** 2026-04-11

**Authors:** Rim Ben Abdallah

**Affiliations:** 1 Institut Pasteur de Tunis, Place Pasteur, Tunisia

General comments : This manuscript describes a rare of primary cutaneous aspergillosis in a neutropenic child. Invasive aspergillosis represents a major cause of morbidity and mortality in immunocompromised patients, particularly in pediatric populations with prolonged neutropenia.

 The topic is therefore of clear clinical interest. The case highlights the diagnostic challenges posed by atypical presentations of invasive fungal infections and emphasizes the importance of early recognition of cutaneous lesions.

 1. Abstract

 - and and improve outcomes” → remove duplicate “and”.

 2. Introduction

 'No evident prior skin trauma' is an important point, but it could be better contextualised by briefly mentioning that PCA is usually associated with skin breaches caused by catheters, dressings or trauma, which makes this case unusual.

 - 5 year- old patient” → correct spacing: 5-year-old patient

 - The abbreviation 'PCA' should be used throughout the remainder of the manuscript after the first definition of primary cutaneous aspergillosis.

 3. Case report :

 -clearer justification of PCA diagnosis : The authors should clarify: whether the ankle lesion was located near an intravenous catheter site, dressing, or skin injury, whether any local predisposing factors were present (skin maceration, adhesive tape, occlusion, trauma)

 - Lack of mycological confirmation from respiratory samples: The authors should explain why respiratory sampling was not performed (e.g. clinical instability).

 - The authors should clarify the exact interval between chemotherapy and the onset of symptoms; the timing of neutropenia and its duration and the interval between the appearance of skin lesions and pulmonary symptoms..

 - If possible, it would be helpful to include other biological elements, such as CRP, and other microbiological analyses to rule out bacterial or viral infection.

 - The reported serum galactomannan index is positive at 0.57. The authors should clarify the positivity cut-off point used.

 - The six-month follow-up is valuable, but the report could be strengthened by including additional details, such as the presence or absence of residual scarring, imaging follow-up of pulmonary lesions and continuation of antifungal prophylaxis.

 4. Discussion

 1. The main limitation of the discussion is the interpretation of the infection as primary cutaneous aspergillosis that has disseminated to the lungs. A diagnosis of PCA requires compelling evidence that the skin lesion occurred prior to systemic involvement.

 In this case, however, pulmonary lesions were detected shortly after the cutaneous lesion, and no mycological confirmation was obtained from respiratory samples.

 The authors should clearly state this limitation in their interpretation of the primary cutaneous origin, and discuss the possibility of secondary cutaneous involvement.

 2. « In this case, the positive GM antigenemia, concomitant with the cutaneous biopsy results and pulmonary imaging, was instrumental in confirming disseminated infection ».

 The role of galactomannan antigenemia in confirming disseminated infection may be overstated, particularly given the borderline index value (0.57) and the absence of microbiological confirmation from respiratory samples. The authors may consider presenting this result as supportive evidence rather than definitive proof of dissemination.

Are enough details provided of any physical examination and diagnostic tests, treatment given and outcomes?

Partly

Is the case presented with sufficient detail to be useful for other practitioners?

Partly

Is sufficient discussion included of the importance of the findings and their relevance to future understanding of disease processes, diagnosis or treatment?

Partly

Is the background of the case’s history and progression described in sufficient detail?

Yes

Reviewer Expertise:

parasitology-mycology

I confirm that I have read this submission and believe that I have an appropriate level of expertise to confirm that it is of an acceptable scientific standard, however I have significant reservations, as outlined above.

# Response

**Published:** 2026-05-20

**Authors:** Yasmine Kalboussi

**Affiliations:** Parasitology-Mycology, Farhat Hached University Hospital of Sousse, Sousse, Sousse, Tunisia

Dear Reviewer,

 Thank you very much for your careful review of our manuscript and for your valuable comments and suggestions. We sincerely appreciate the time and effort you dedicated to evaluating our work.

 We have carefully considered all of your remarks and have revised the manuscript accordingly. The changes made in response to your comments have helped us improve the clarity, quality, and scientific rigor of the article.

 Please find below our point-by-point responses to your comments:

1. **Abstract :** ” - and and improve outcomes” → remove duplicate “and”.

Response: We have deleted ‘and ‘ line 72

1.  
**Introduction**

- **'No evident prior skin trauma'** is an important point, but it could be better contextualised by briefly mentioning that PCA is usually associated with skin breaches caused by catheters, dressings or trauma, which makes this case unusual.
Response: We appreciate this suggestion. In response, we have added ‘PCA is typically associated with breaches in skin integrity caused by catheters, dressings, or trauma, making the present case particularly unusual’

 

- **5 year- old patient”** → correct spacing: 5-year-old patient

Response: We have correted by adding a space ‘5-year-old patient’ Line 87

  

- **The abbreviation 'PCA'** should be used throughout the remainder of the manuscript after the first definition of primary cutaneous aspergillosis.

Response : Thank you for this valuable comment. The abbreviation “PCA” was already defined for primary cutaneous aspergillosis, and we have ensured that the abbreviation is used consistently throughout the remainder of the manuscript.

1. **Case report :**

- **Clearer justification of PCA diagnosis :** The authors should clarify: whether the ankle lesion was located near an intravenous catheter site, dressing, or skin injury, whether any local predisposing factors were present (skin maceration, adhesive tape, occlusion, trauma.

Response: Thank you for highlighting this issue. We agree that clarification of potential local predisposing factors is essential for supporting the diagnosis. We have revised the manuscript to specify that no intravenous catheter, dressing, or local traumatic lesion was present at the site of the ankle lesion, and no obvious predisposing skin condition (such as maceration or occlusion) was identified.

 We added ‘with no evidence of intravenous catheterization, local trauma, dressing, or other identifiable predisposing skin condition at the lesion site.’

  

- **Lack of mycological confirmation from respiratory samples:** The authors should explain why respiratory sampling was not performed (e.g. clinical instability).

Response :  Thank you for this important comment. We acknowledge the absence of respiratory mycological sampling. In this case, the patient was thrombocytopenic and clinically fragile, which limited the feasibility of invasive respiratory procedures. We have clarified this point in the revised manuscript.

 We added ‘Respiratory sampling was not performed due to the patient’s thrombocytopenia and clinical fragility’

- **The authors should clarify the exact interval **between chemotherapy and the onset of symptoms; the timing of neutropenia and its duration and the interval between the appearance of skin lesions and pulmonary symptoms..

Response : Thank you for this helpful comment. The interval between chemotherapy administration and the onset of symptoms, as well as the timing and duration of neutropenia, were already specified in the manuscript. In response to your suggestion, we have now added clarification regarding the interval between the appearance of the skin lesion and the subsequent pulmonary symptoms to further improve the clarity of the clinical timeline.

 We added ‘When a mild cough appeared seven days after the onset of the cutaneous lesion,’

- **If possible, it would be helpful to include other biological elements**, such as CRP, and other microbiological analyses to rule out bacterial or viral infection.

Response : Thank you for this helpful suggestion. We have added a general description of the inflammatory marker assessment and microbiological investigations performed,

 We added ‘Biological investigations demonstrated elevated inflammatory markers, including C-reactive protein (CRP), and bacterial and viral microbiological workup was negative.’

- **The reported serum galactomannan index** is positive at 0.57. The authors should clarify the positivity cut-off point used.

Response

 Thank you for this important comment. We have now specified the positivity cut-off used for the serum galactomannan assay (Platelia Aspergillus, Bio-Rad).

 We added ‘, using the manufacturer-recommended positivity cut-off of 0.5.‘

1. **Discussion**

- **The main limitation of the discussion** is the interpretation of the infection as primary cutaneous aspergillosis that has disseminated to the lungs. A diagnosis of PCA requires compelling evidence that the skin lesion occurred prior to systemic involvement.
  In this case, however, pulmonary lesions were detected shortly after the cutaneous lesion, and no mycological confirmation was obtained from respiratory samples.
  The authors should clearly state this limitation in their interpretation of the primary cutaneous origin, and discuss the possibility of secondary cutaneous involvement.

Reponse :

 Thank you for this insightful comment. We fully acknowledge this limitation regarding the interpretation of the disease sequence and the potential origin of the infection. We have revised the manuscript accordingly to explicitly state that, although our findings support a primary cutaneous origin, the possibility of secondary cutaneous involvement in the context of systemic disease cannot be completely excluded.

 We added ‘However, we acknowledge the limitation that a secondary cutaneous involvement in the context of systemic disease cannot be completely excluded.’

- **« In this case, the positive GM antigenemia,** concomitant with the cutaneous biopsy results and pulmonary imaging, was instrumental in confirming disseminated infection ».
  The role of galactomannan antigenemia in confirming disseminated infection may be overstated, particularly given the borderline index value (0.57) and the absence of microbiological confirmation from respiratory samples. The authors may consider presenting this result as supportive evidence rather than definitive proof of dissemination

Reponse

 Thank you for this pertinent remark. We agree that the contribution of galactomannan antigenemia in this case should not be overstated, particularly given the borderline index value (0.57).

 We added ‘In this case, the galactomannan antigenemia (index 0.57) was borderline and not sufficient on its own to confirm disseminated infection.’

 We hope that the revised version adequately addresses your concerns and meets the journal’s expectations.

 Thank you again for your constructive feedback.

 Sincerely,

 Yasmine Kalboussi
